# Histone H3 N-terminal acetylation sites especially K14 are important for rDNA silencing and aging

**DOI:** 10.1038/srep21900

**Published:** 2016-02-24

**Authors:** Heng-hao Xu, Trent Su, Yong Xue

**Affiliations:** 1 Jiangsu Key Laboratory of Marine Pharmaceutical Compound Screening, Huaihai Institute of Technology, Lianyungang 222005, China; 2 Co-Innovation Center of Jiangsu Marine Bio-industry Technology, Lianyungang, 222005, China; 3 Department of Biological Chemistry, David Geffen School of Medicine, University of California, Los Angeles, CA 90095, USA

## Abstract

Histone variants and histone modifications are essential components in the establishment and maintenance of the repressed status of heterochromatin. Among these histone variants and modifications, acetylation at histone H4K16 is uniquely important for the maintenance of silencing at telomere and mating type loci but not at the ribosomal DNA locus. Here we show that mutations at H3 N-terminal acetylation site K14 specifically disrupt rDNA silencing. However, the mutant ion at H3K14R doesn’t affect the recruitment of Pol II repressor RENT (regulator of nucleolar silencing and telophase exit) complex at the rDNA region. Instead, the CAF-1(chromatin assembly factor I) subunit Cac2 level decreased in the H3K14R mutant. Further experiments revealed that the single mutation at H3K14 and multi-site mutations at H3 N-terminus including K14 also delayed replication-depend nucleosome assembly and advanced replicative life span. In conclusion, our data suggest that histone H3 N-terminal acetylation sites especially at K14 are important for rDNA silencing and aging.

Heterochromatin makes up a large percentage (as much as 30%) of chromatin in higher eukaryotes including humans and is important for proper chromosome segregation and genome stability. Disruption of heterochromatin can impair normal gene transcription and lead to the development of different diseases including cancer^1^. Yeast has provided an important model system with which to understand major conserved processes in the formation of heterochromatin. In the budding yeast *Saccharomyces cerevisiae*, silent heterochromatic DNA domains occur at telomeres, the silent mating type loci (HML and HMR), and the ribosomal DNA (RDN1) repeats^2^. Histone variants and histone modifications are very important for the establishment and maintenance of transcriptionally repressive heterochromatin. Among the different histone variants and modifications, it is well studied that the acetylation status of histone H4 lysine 16 is critical for the heterochromatin silencing at telomere and mating type loci through the physical interaction between H4K16 and Sir3^3^,^4^. However, compared to telomere and mating type loci, the role of histone modifications in rDNA silencing is not well understood.

In *Saccharomyces cerevisiae*, the ribosomal DNA (RDN1) locus on Chr. XII is composed of ~150 tandem rDNA repeats encoding the rRNAs. Each repeat contains a Pol I-transcribed 35S rRNA gene and a non-transcribed spacer (NTS) which is divided into NTS1 and NTS2 by the Pol III-transcribed 5S rRNA gene. Despite high levels of transcription by Pol I and Pol III in the rDNA locus, Pol II transcribed genes integrated into the rDNA are silenced (referred to rDNA silencing)^5^,^6^,^7^. The silenced chromosomal region at rDNA is essential for repression of genetic recombination^8^,^9^,^10^ and extension of replicative life span (reviewed in Guarente)^11^. The silencing of Pol II transcripts that occurs in rDNA NTS1 and NTS2 relies on the nucleolar RENT (regulator of nucleolar silencing and telophase exit) complex, which consists of Sir2, Net1 and Cdc14^12^,^13^. The maintenance of repressed status in the NTS region also requires multiple DNA replication and chromatin-modulating factors such as Set1, a protein that methylates histone H3 at lysine 4^14^. Also, a systematic screen in histone mutants showed that 77% of histone H3 N-terminal tail deletions have LRS (loss of rDNA silencing) phenotype whereas only 33% and 8% showed an effect at telomeres and HMR, indicating the important role of the H3 tail in rDNA silencing^15^. However, the reported range of the lead plate expression assay (−2 to + 2) in the screening does not quantify the large differences in expression and does not clarify how the H3 tail regulates rDNA silencing.

In this study, we investigated the role of histone H3 N terminal acetylation in rDNA silencing by measuring the expression of silencing reporter gene *MET15,* which was integrated into the RDN1 locus. Surprisingly, we found that among H3 N terminal acetylation residues (K9, K14, K18, K23, and K27), K14 is uniquely important for rDNA silencing. However, the LRS mutation H3K14R does not affect RENT complex recruitment. Instead, the recruitment of chromatin assembly factor (CAF-1) subunit Cac2 is decreased in H3K14R mutant. Further experiments revealed that H3K14 acetylation regulates replication-depend nucleosome assembly and replicative aging. Taken together, our data indicate that histone H3 N-terminal acetylation sites especially at K14 are important for rDNA silencing and aging, possibly through replication-dependent nucleosome assembly factor CAF-1.

## Results

### Histone H3K14 acetylation is uniquely important for rDNA silencing

**Figure 1:**
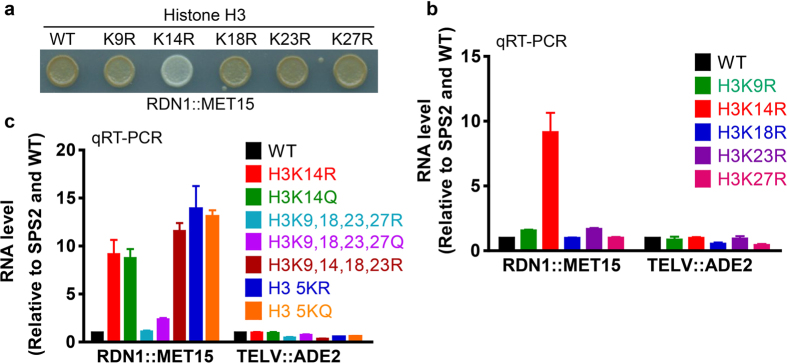
Histone H3 N terminal acetylation site mutations especially K14 affect rDNA silencing. (**a**) Color assay showing the phenotypes of wide-type (WT) and H3 mutants on rDNA silencing. The reporter gene *MET15* was integrated in the rDNA locus to show the silenced status (brown) and depressed status (white). (**b,c**) qRT-PCR of *MET15* at RDN1 and *ADE2* at TELV in strains containing wide-type or mutated histone H3. H3 5KR refers to H3K9,14,18,23,27R and H3 5KQ refers to H3K9,14,18,23,27Q. Cells were grown in YPD and collected in log phase. Data are presented as mean ± standard error of mean (SEM).

The analysis of the Histone Systematic Mutation Database indicates that the H3 tails acetylation is involved in RDN1 silencing^16^. However, it is hard to distinguish the difference between the individual residue mutants and the redundancy of these mutaitons based on the reported range of the lead plate expression assay (−2 to + 2). To determine which lysine residues are primarily involved and/or whether their function are redundantly involved in RDN1 silencing, we used RT-PCR to examine the expression of *MET15* reporters at the RDN1 locus in nested H3 N terminal single and multiple amino acid substitutions at five H3 acetylation sites. Arginine (R) and glutamine (Q) substitution were used to mimic unacetylated and acetylated form of lysine (K), respectively. Surprisingly, we found that among the H3 acetylation site substitution mutants (K9R, K14R, K18R, K23R, and K27R), only the K14R mutant has highly expressed *MET15* (Fig. 1a,b). Similarly, *MET15* was also highly expressed in the K14Q mutant compared to other glutamine substitutions (K9Q, K18Q, K23Q and K27Q) mutants as seen in colony color silencing assays (Figure S1). These data indicate that both the H3K14 acetylation and deacetylation are specifically required to maintain RDN1 silencing.

To further investigate the specific role of H3K14 in RDN1 silencing, we measured rDNA silencing in mutants containing multiple amino acid substitutions at H3 N-terminal tail acetylation sites. As shown in Fig. 1c, the silent status of MET15 was still maintained in the H3 K9,18,23,27R mutant (wide type K14) and there was some weak induction of MET15 in the H3 K9,18,23,27Q mutant (wide type K14). However, the induction of *MET15* in K14R and K14Q mutants was much higher, to a level close to H3K9,14,18,23R, H3 5KR (K9,14,18,23,27R) and H3 5KQ (K9,14,18,23,27Q) mutants. At the same time, all these mutants did not induce the expression of another reporter gene *ADE2,* which was integrated into the telomeric region at chromosome V. Taken together, our data indicate that H3 N-terminal tail acetylation sites especially K14 are important for rDNA silencing.

### H3K14 acetylation does not affect RENT complex recruitment at RDN1 region

To investigate the possible mechanism of by which H3 tail acetylations, especially at K14, regulate rDNA silencing, we first asked whether H3 tail mutants affect Fob1 recruitment at rDNA region. Fob1 is a nucleolar protein that binds the rDNA replication fork barrier site (RFB) and is required to repress Pol II transcription around RFB at NTS1. As shown in Figure S2, Fob1 was specifically enriched at the RFB site but its level doesn’t decrease in H3 mutants. These data suggest that H3K14R does not suppress rDNA silencing through Fob1.

**Figure 2:**
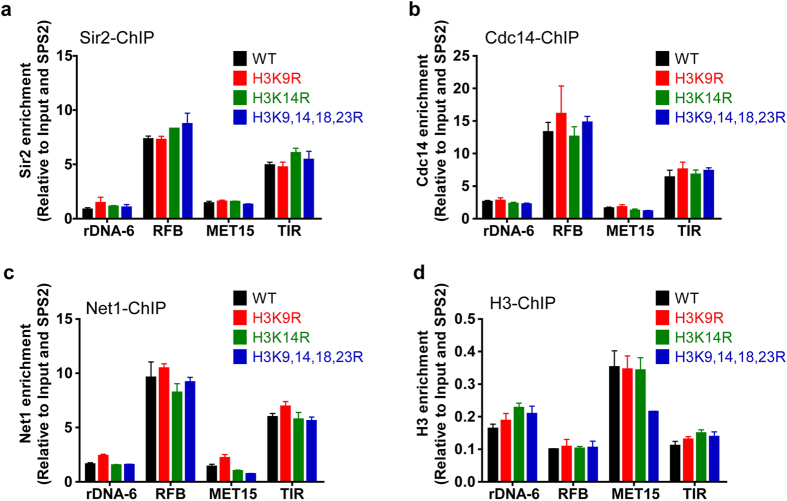
RENT complex recruitment at rDNA region is not affected in H3 mutants. (**a–c**) ChIP of RENT complex Sir2 (**a**), Cdc14 (**b**) and NET1 (**c**) in H3 mutants. (**d**) ChIP of histone H3 in H3 mutants. The DNA from IP was normalized to Input and SPS2.

RENT complex, which includes Sir2, cdc14 and Net1, also has been shown to repress the Pol II transcribed region of NTS1 and NTS2 inside the rDNA region^13^. The recruitment of RENT complex requires localization of Fob1 at NTS1 and UAF complex (upstream activator factors) at NTS2^12^,^17^. To ask whether the H3K14R mutant affects RENT complex recruitment at the RDN1 locus, we measured the enrichment of Sir2, Cdc14 and Net1 in H3 mutants. As shown in Fig. 2, Cdc14, Sir2 and Net1 were enriched at the RFB in NTS1 and TIR (Pol I transcription initiation region) in the NTS2 region. This observation is consistent with previous result^12^. However, the RENT complex levels did not decrease in the H3 mutants as compared to WT at these regions. And there was no significant difference of the presence of RENT complex in the *MET15* coding region between WT and H3K14R. All these data indicate that H3K14R does not disrupt rDNA silencing through Fob1 and RENT complex.

### The recruitment of CAF-1 subunit Cac2 is decreased in the H3K14R mutant

**Figure 3:**
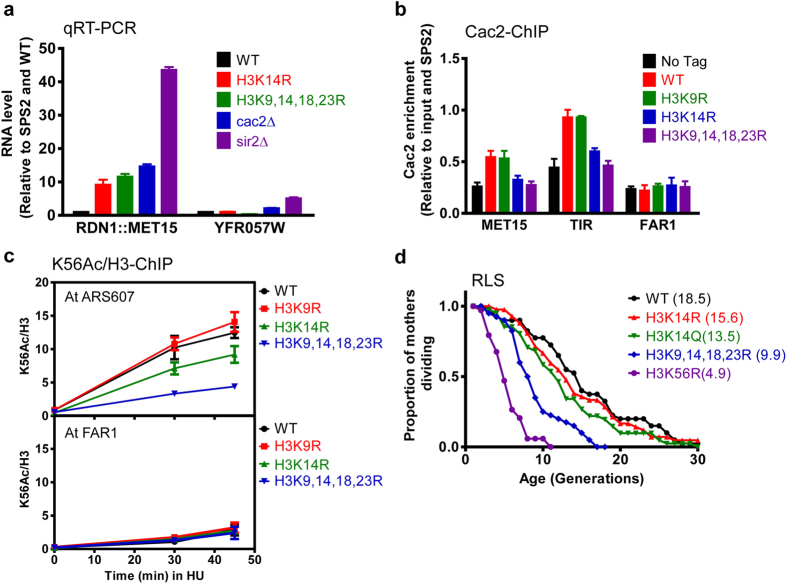
Histone H3 N terminal acetylation sites mutations especially at K14 affect nucleosome assembly and replicative aging. (**a**) qRT-PCR of *MET15* at RDN1 and *YFR057W* at native TELVIR in WT and histone H3 mutations, *cac2*∆ and *sir2*∆ strains. (**b**) ChIP of CAF-1 complex subunit Cac2 in H3 mutants. TAP tag was fused to CAC2 to pull down the Cac2. (**c**) The deposition of new histone (marked as H3K56Ac level) at replicating DNA in H3 mutant cells. The ChIP DNA was analyzed using primers that amplify the replication origin ARS607 or FAR1 (a fragment 14 kb away from ARS607, ARS607 + 14 kb). The ratio of H3K56Ac ChIP signal over that of H3 was calculated. (**d**) The replicative life span of H3 mutants in RMY200 background.

Recent reports showed that H3 N terminal acetylation is required for the replication-dependent nucleosome assembly by CAF-1 (chromatin assembly factor 1 including three subunits Cac1, Cac2 and Cac3) which has been shown to also have defects in rDNA silencing^18^,^19^. This suggests that CAF-1 may be involved in rDNA silencing regulated by H3 tail acetylation. To test this, we first checked the *MET15* level in deletion mutant of CAF-1 subunit *CAC2* and the H3 tail mutants. As shown in Fig. 3a, the deletion of *CAC2* derepressed rDNA silencing to a similar extent compared to the H3 5KR mutant. However, deletion of *SIR2* had a stronger phenotype. This supports the idea that Cac2 may be involved in the rDNA silencing regulated by H3 tail acetylation. To ask whether the H3 tail acetylation is required for the recruitment of Cac2 at the *MET15* gene and the RDN1 locus, we fused Cac2 with TAP tag and measured its level in the H3 mutants. As shown Fig. 3b, Cac2 level decreased both at the MET15 gene and RDN1 locus TIR in H3K14R and H3K9,14,18,23R mutants, but not in H3K9R. And there was no enrichment of Cac2 at FAB1 (14 kb away from ARS607) used as a negative control. These data suggest that H3 tail acetylations especially at K14 are required to recruit Cac2 at ribosome DNA locus and regulate rDNA silencing.

The decreased Cac2 level in H3K14R indicates that K14 acetylation alone may also specifically regulate replication depend nucleosome assembly. To test this, we used H3K56 acetylation as a marker to measure the newly-incorporated histone levels when cells enter the S phase in the H3 mutants as previously described^19^. As shown in Fig. 3c, H3K9,14,18,23R had a strong defect in the incorporation of new histone as compared to WT and H3K9R at ARS607, which is similar to H3 5KR^19^. It was interesting that H3K14R, to a weaker effect than H3K9,14,18,23R, also affects nucleosome assembly. In summary, our data suggest that H3K14 acetylation may have a dominant role among the five H3 tail acetylation sites to regulate nucleosome assembly through CAF-1 complex.

### H3K14 acetylation is important for replicative aging

To determine the consequence of defects in replication-coupled nucleosome assembly, we measured replicative life span in the H3 mutants. H3K56 acetylation dependent nucleosome assembly by Asf1 had been shown to be involved in the regulation of replicative aging^20^. Here we show that H3 tail acetylation sites especially K14 are also involved in replication-depended nucleosome assembly. Therefore, we were interested in whether H3 tail acetylations including K14 acetylation also regulate replicative aging. To test this, we measured replicative life span as previously described^21^ in the WT strain and strains containing mutations at the H3 N terminus. The H3K56R mutant was used as a negative control. As shown in Fig. 3d, the H3K14Q single mutation significantly advanced replicative life span, whereas the H3K14R mutation showed only a marginal effect. This effect of H3K14 mutants in replicative life span was similar to that of H4K16 mutants as previously reported^22^. The multi-residues mutations in H3K9,14,18,23R further shortened the replicative life span and K3K56R had the strongest effect. In the H3 mutants, the trend of defects in replicative aging was similar to that of the defects in nucleosome assembly, and was consistent with Feser’s work showing that the *ASF1* mutant had a stronger defect in replicative aging than cac1 mutant^20^.

## Discussion

In this study we report that H3K14 acetylation is uniquely important in the regulation of ribosome DNA silencing. This regulation is not mediated through repressor Fob1 or RENT complex. The unique role of H3K14 acetylation in rDNA silencing makes it comparable to the role of H4K16 acetylation in regulation of telomere and mating type loci silencing. The facts that sir2Δ mutant but not *hst1*Δ*, hst2*Δ double mutant disrupt rDNA silencing^23^ and increased H3 acetylation including K14 in sir2Δ mutant^12^ raise the possibility that H3K14 acetylation may be targeted by histone deacetylase Sir2 within the RENT complex. In fact, compared to H3K14, H3K9 mutation not only has a weak effect on *RDN1* silencing but H3K9 acetylation is also a weak target for deacetylation by the RENT complex *in vitro*^24^. However, whether H3K14 acetylation is specifically targeted by Sir2 within the RENT complex requires further investigation.

Importantly, our data reveal that H3K14 acetylation is especially crucial and redundant with other H3 tail acetylations for the replication-dependent nucleosome assembly through CAF-1. Burgess *et al.* reported that the major H3 N-terminal acetyltransferase Gcn5 is required for replication-dependent nucleosome assembly through CAF-1^19^. Furthermore, Wittner *et al.* reported the new histones are deposited at the rDNA region only during replication and not during transcription. Additionally, replication-dependent nucleosome assembly is essential in converting rRNA genes into the closed chromatin states^25^. In accordance with our results, we speculate that H3 N-terminus acetylation especially at K14 regulate rDNA silencing through replication dependent nucleosome assembly by CAF-1 complex. This is supported by the findings that Gcn5 and CAF-1 subunit Cac1 both repress Pol II transcription at rDNA^18^,^26^,^27^. Furthermore, the fact that both H3K14 acetylation and deacetylation are important for rDNA silencing also indicates the involvement of nucleosome assembly which is stimulated by the acetylation-deacetylation cycle. However, it is still possible that H3K14 acetylation regulates rDNA silencing through other factors. For example, chromatin structure remodeling (RSC) complex directly interacts with H3K14 acetylation and its mutants display defects in rDNA silencing. H3 tail acetylations are also involved in modulating the ATP-dependent nucleosome remodeling by SWI/SNF^28^ whose mutants also show defects in RND1 silencing. Another unique role of H3K14 acetylation in nucleosome eviction^29^ may also be connected to the specific role of H3K14 acetylation in rDNA silencing. The role and mechanism of nucleosome assembly and remodeling complex in rDNA silencing is still not well understood and needs further investigation.

Histones and posttranslational histone modifications regulate numerous cellular processes including aging. However, the role of histones and histone modifications in aging is not well studied. Previously only H3K56 and H4K16 acetylation have been shown to regulate replicative aging through nucleosome assembly and maintenance of intact telomeric chromatin, respectively. Here we showed that H3 tail acetylation is involved in the regulation of replicative aging. The advanced replicative life span in H3 mutants may mainly be affected through defects in nucleosome assembly instead of rDNA silencing or maintenance of intact telomeric chromatin. This is supported by the facts that the either H3K14 single mutant (H3K14R) or the H3 tail multiple mutant (H3K9,14,18,23R) does not affect the recombination rate in rDNA region (data not shown), and H3 tail mutations do not disrupt telomere silencing. Additionally, yeast lacking histone chaperone Asf1 or acetylation of histone H3 on lysine 56 has shortened life span because of the defects in nucleosome assembly and decreased histone levels^20^. Gcn5, which acetylates H3 N terminus *in vivo*^30^ and promotes replication-coupled nucleosome assembly^31^, also affects replicative aging^27^. Taken together, we speculate that the new role of H3 tail acetylation especially at K14 in replicative aging is caused by deficiencies in replication-dependent nucleosome assembly.

In summary, we have shown that histone H3 N-terminal tail acetylation, especially at K14, is important for the maintenance of rDNA silencing, replicative nucleosome assembly, and replicative aging.

## Methods

### Yeast strains, Plasmids and Media

Yeast strains and plasmids used in this work were described in Table S1. Standard yeast media and manipulations were used.

### Colony color silencing assays

Silencing of an MET15 reporter gene integrated at rDNA was examined by growth of cells on MLA plates as described previously^7^. Briefly, Strains to be tested were patched onto YPD and grown overnight. The cells were diluted to OD_600_ 0.2 and collected at OD_600_ around 1. Then cells were spotted in MLA plates and incubated at 30 °C for 2–3 days before photographing.

### qRT-PCR

qPCR were performed as described previously^32^. Briefly, total RNA was extracted using the hot acid phenol extraction method from log phase cells. The extracted RNA samples were treated with DNase I (Qiagen), purified and reverse-transcribed using random primers and M-MLV reverse transcriptase (Invitrogen). qPCR of the cDNA was performed on an Applied Biosystems 7500 Real-Time PCR system using the Maxima SYBR Green qPCR Master Mix (Fermentas) according to the manufacturer’s instructions. The ΔΔCt method was used to calculate relative gene expression levels^33^.

### ChIP

Log phase cells were cross linked with 1% formaldehyde and standard ChIP assays were performed as described previously^32^. 50 μl of lysate were used per ChIP assay with the following amounts of antibodies: 3 μl Sir2 (in house/480), 10 μl Net1 (Santa Cruz/sc-27758), 10 μl Cdc14 (Santa Cruz/sc-12045), 2.5 μl K56Ac (in house/665), 3 μl Myc (Roche/9E10). DNA was extracted from the immunoprecipitated chromatin using the fast ChIP method^34^. For the H3 ChIP, 100 μl of chromatin was diluted with 800 ml of 140 μM lysis buffer^35^ and incubated overnight along with 1 μg of anti-H3 antibody (Abcam/Ab1791) and 50 ml of 50% protein-A sepharose beads. Chromatin from the H3 ChIP assay was heat denatured at 95 °C for 25 min and purified using the QIAquick PCR Purification Kit (QIAGEN). For HU treatment, log phase cells were first arrested by alpha factor for 4 hours and release in 0.2 M HU. Cells were collected at different time and ChIP was performed using antibody against H3 K56Ac and H3 as described before.

### Measurement of replicative Life Span

Replicative life span was determined as described^21^. Briefly, WT and H3 tail mutation strains were grown on YPD Plates. Life span was determined by scoring the number of daughter cells produced by each mother cell before cessation of cell division. Daughter cells were separated from mother cells by microdissection.

## Additional Information

**How to cite this article**: Xu, H.- *et al.* Histone H3 N-terminal acetylation sites especially K14 are important for rDNA silencing and aging. *Sci. Rep.* **6**, 21900; doi: 10.1038/srep21900 (2016).

## Supplementary Material

Supplementary Information

Supplementary Dataset
