# Supplementary material for: Histone H3 N-terminal acetylation sites especially K14 are important for rDNA silencing and aging
Source: Sci Rep. 2016 Feb 24;6:21900. doi: 10.1038/srep21900 (PMC4764821; doi:10.1038/srep21900)

Supplementary information

Histone H3 N-terminal acetylation sites especially K14 are important for rDNA silencing and aging

Heng-hao Xu[1,2], Trent Su[3], Yong Xue[1,3,*],

[1] Jiangsu Key Laboratory of Marine Pharmaceutical Compound Screening, Huaihai Institute of Technology, Lianyungang 222005, China.

[2] Co-Innovation Center of Jiangsu Marine Bio-industry Technology, Lianyungang, 222005, China.

[3] Department of Biological Chemistry, David Geffen School of Medicine, University of California, Los Angeles, CA 90095, USA

Supplementary Table S1. Lists of yeast strains and plasmids.
Available as separate MS Excel file

Supplementary Figure S1 Histone H3 N terminal acetylation site glutamine mutations, especially K14, affect rDNA silencing. The image is the color assay result showing the phenotypes of wide-type (WT) and H3 mutants on rDNA silencing. The reporter gene *MET15* was integrated in the rDNA locus to show the silenced status (brown) and depressed status (white).

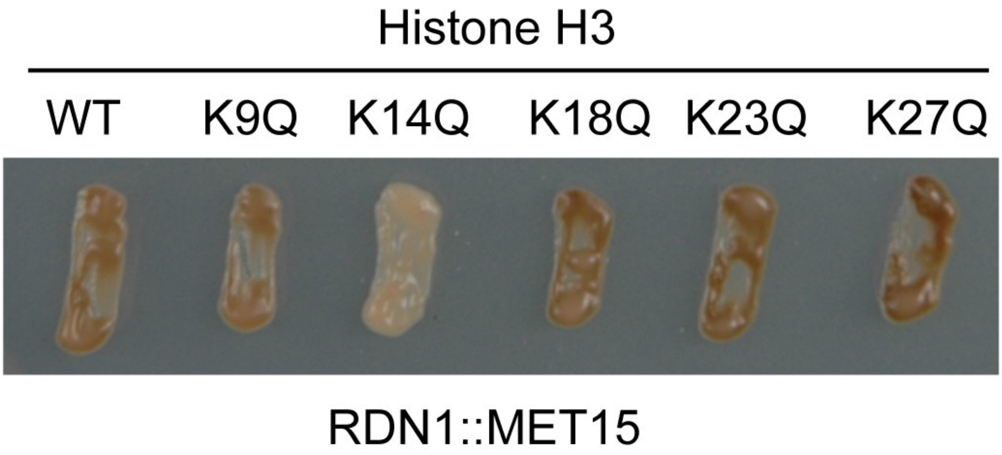

Supplementary Figure S2 Fob1 recruitment at rDNA region is not affected in H3 mutants. The DNA from Fob1 IP in H3 mutants were normalized to Input and SPS2.

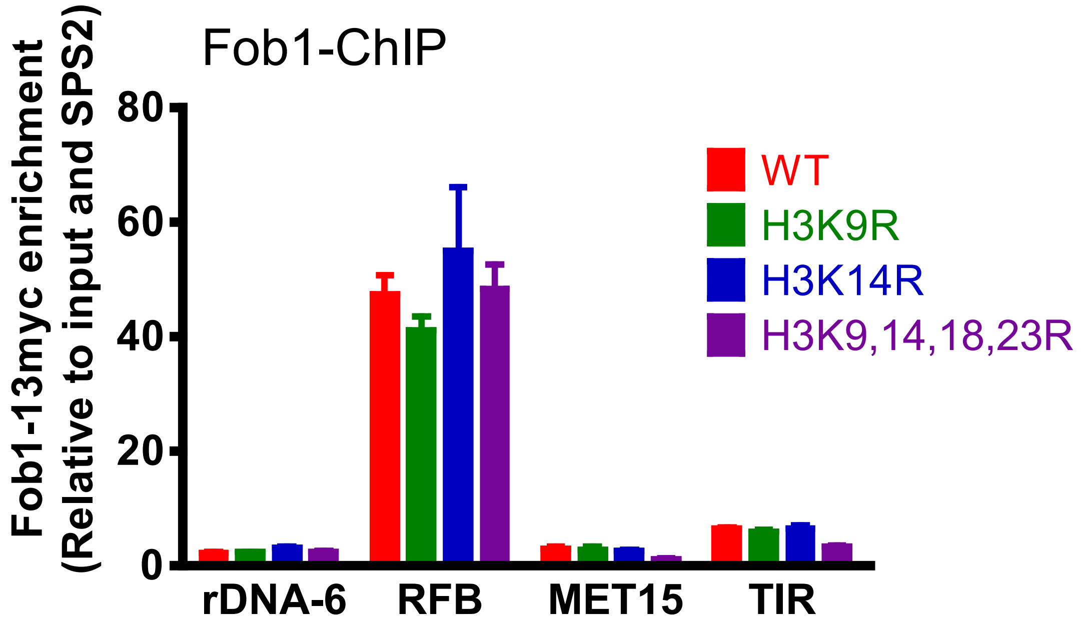

Supplement: Supplementary Information [file srep21900-s1.pdf]
